# Research on Human-Robot Collaboration Method for Parallel Robots Oriented to Segment Docking

**DOI:** 10.3390/s24061747

**Published:** 2024-03-08

**Authors:** Deyuan Sun, Junyi Wang, Zhigang Xu, Jianwen Bao, Han Lu

**Affiliations:** 1 School of Mechanical Engineering, Shenyang University of Technology, Shenyang 110870, China; sundeyuan@sia.cn; 2 Shenyang Institute of Automation, Chinese Academy of Sciences, Shenyang 110016, China; jywang@sia.cn (J.W.); baojianwen1@sia.cn (J.B.); luhan@sia.cn (H.L.); 3 State Key Laboratory of Robotics, Shenyang Institute of Automation, Chinese Academy of Sciences, Shenyang 110016, China; 4 Institutes for Robotics and Intelligent Manufacturing, Chinese Academy of Sciences, Shenyang 110169, China; 5 University of Chinese Academy of Sciences, Beijing 100049, China

**Keywords:** parallel robot, admittance control, fractional-order control, robust control, human-robot collaboration

## Abstract

In the field of aerospace, large and heavy cabin segments present a significant challenge in assembling space engines. The substantial inertial force of cabin segments’ mass often leads to unexpected motion during docking, resulting in segment collisions, making it challenging to ensure the accuracy and quality of engine segment docking. While traditional manual docking leverages workers’ expertise, the intensity of the labor and low productivity are impractical for real-world applications. Human-robot collaboration can effectively integrate the advantages of humans and robots. Parallel robots, known for their high precision and load-bearing capacity, are extensively used in precision assembly under heavy load conditions. Therefore, human-parallel-robot collaboration is an excellent solution for such problems. In this paper, a framework is proposed that is easy to realize in production, using human-parallel-robot collaboration technology for cabin segment docking. A fractional-order variable damping admittance control and an inverse dynamics robust controller are proposed to enhance the robot’s compliance, responsiveness, and trajectory tracking accuracy during collaborative assembly. This allows operators to dynamically adjust the robot’s motion in real-time, counterbalancing inertial forces and preventing collisions between segments. Segment docking assembly experiments are performed using the Stewart platform in this study. The results show that the proposed method allows the robot to swiftly respond to interaction forces, maintaining compliance and stable motion accuracy even under unknown interaction forces.

## 1. Introduction

Assembling large-scale aerospace equipment involves cabin segment docking, a pivotal aspect in manufacturing that directly impacts product precision and stability [1]. Conventional manual docking methods, requiring high precision in terms of worker operation and involving labor-intensive processes, fall short of meeting practical application demands [2]. Parallel robots boast advantages such as a high load capacity, precise positional control, and low end-effector inertia, making them ideal for assembling large or heavy components [3]. Therefore, employing parallel robots for cabin segment docking and developing a high-precision, high-efficiency docking system is of significant importance.

Aerospace cabin segments, which are usually large and heavy, require precise docking without collisions or pressure to safeguard against damage to the segments and internal seals during docking procedures [4]. Achieving fully automated, high-precision and high-reliability docking is challenging as it often depends on manual adjustments during the cabin segment docking process to ensure precision and reliability [5]. Maximizing the strengths of robots and humans, merging human expertise with parallel robots’ precise positioning, and employing collaborative human-robot methods through parallel robots for segment docking offer an effective and practical path to accomplish efficient, precise, and reliable docking procedures.

Space cabin segments require docking precision in the tens of micrometers while their substantial mass causes significant inertia during movement. Precise adjustments and significant inertial effects often lead to collisions during docking, posing a greater risk of segment damage [6]. Resolving this problem may require implementing a high-compliance strategy for Human-robot collaboration. This approach entails real-time adjustments to the robot’s movements, compensating for the extra motion induced by inertia in the docking process, effectively averting unforeseen movements that could result in collisions. The present research mainly concentrates on perceiving operator intentions using diverse methods to attain compliance in human-robot collaboration (HRC) [7]. Elisa Prati et al. introduced a structured approach concentrating on user experience (UX), devising multiple interaction design tools to scrutinize user experience and human-robot dialogues [8]. V. Duchaine et al. equipped the end effector of a three-degree-of-freedom parallel robot with a pen, enabling operators to collaboratively guide the robot through maze-like paths [9]. In their prior research, they finetuned the robot’s control rules by interpreting human intentions [10]. A. Cherubini et al. employed a GA-BP neural network to independently learn the correlation between input parameters and output velocities, maintaining this unknown correlation within the network structure. This allowed for the continuous prediction of operator intention information to derive the operator’s intended actions [11]. Gao Xiaoshan et al. suggested a hybrid recursive neural network structure utilizing deep learning to allow for robots to discern human intentions in collaborative tasks [12]. The present research solely categorizes and recognizes operator intentions by interaction force, adjusting robot control parameters accordingly. However, these studies lack an exact, quantifiable link between adjustment strategies and interaction forces. Hence, it is crucial to further explore the correlation between interaction forces and adjustment strategies to achieve precise human-parallel robot collaboration for cabin segment docking.

Adapting the robot’s movements to prevent segment collisions during docking necessitates rapid responses to interaction forces in collaboration. However, the closed-loop structure of parallel robots induces coupled disturbance torques arising from interconnected inertia among their drive axes. This leads to control overshoot and vibrations during motion control, significantly affecting the robot’s dynamic response [13]. G. A. Leonov et al. examined motion stability concerns related to the Stewart platform, assessing their impact on control delays in practical applications [14]. Shan et al. performed a dynamic coupling torque analysis using joint space dynamic models, defining the dynamic coupling strength coefficient. They analyzed dynamic coupling characteristics based on this coefficient, outlining the variation pattern within the necessary motion trajectories [15]. He Jingfeng et al. formulated the comprehensive dynamic model of a six-degrees-of-freedom parallel robot, evaluating channel coupling in various typical robot poses [16]. The cited literature did not explore the impact of coupling relationships on control algorithms or suggest strategies to improve the response performance of parallel robot control algorithms. Consequently, improving the control precision and response speed in human-robot collaboration using parallel robots warrants further investigation.

In the collaborative docking of segments, parallel robots bearing heavy aerospace segments experience unknown interaction forces, significantly influencing their motion stability. Robot instability can result in unforeseen movements, hence affecting assembly precision [17]. Thus, in human-robot collaboration, the robot’s motion controller generally requires robust control integration. Multiple studies have proposed diverse robust control strategies. For example, Zhang Tie et al. introduced a robust three-dimensional arm force estimation model, R3DNet, based on a hybrid deep learning network, to establish a stable interaction interface, achieving a dependable human-robot collaborative performance [18]. Ge Dongsheng et al. introduced the compliance dynamic system (CDS), a novel force control strategy. They rigorously validated CDS’s stability using the Lyapunov stability theory, ensuring an accurate force control performance [19]. Sehun K et al. presented performance enhancement after stability execution (PESE), a novel robust interaction control strategy. PESE guarantees robust, interactive stability in uncertain environments and ensures an effective contact performance [20]. Mujica Martín et al. employed robust control on a KUKA robot, empowering the robot end-effector to manage variable and unknown loads for successful human-robot collaboration [21]. Pei Jiufang et al. suggested a robust fuzzy sliding mode control approach based on impedance, enhancing the robot system’s ability to resist disturbances [22]. The aforementioned studies primarily concentrated on robust control research for conventional serial robots. However, for parallel robots, their closed-loop structure introduces indeterminate Coriolis and coupling terms in the dynamic model, rendering the robustness analysis more challenging in the presence of uncertainties. Consequently, attaining robust dynamic motion control remains an unresolved challenge when precise dynamic models for parallel robots are unattainable.

Focusing on the current human-robot collaboration (HRC) strategy for parallel robots, issues arise regarding stability, the lack of flexibility, and the slow response speed during collaboration. This paper proposes a fractional-order variable damping admittance control (FVDAC) that adjusts the damping matrix within the admittance control system according to the interaction force exerted by the operator. Additionally, it establishes a functional relationship between the interaction force and the damping matrix, enabling the Stewart platform to discern the operator’s intentions during collaborative assembly, and dynamically adjusting its velocity. Concurrently, fractional-order control significantly enhances the controller’s response performance. This can be used to tackle the issue of inadequate motion stability in parallel robots caused by unknown interaction forces during collaboration, consequently impacting assembly quality. This paper introduces an inverse dynamic robust control mechanism to enhance the motion controller’s robustness against unknown interaction forces.

The rest of this paper is divided as follows: Section 2 outlines the application scenarios, and Section 3 delineates the control algorithms utilized in the preceding human-machine collaboration. Following this, Section 4 outlines the experimental results and ensuing discussions. Lastly, Section 5 offers the conclusion and potential perspectives.

## 2. Application Scenario Description

**Figure 1:**
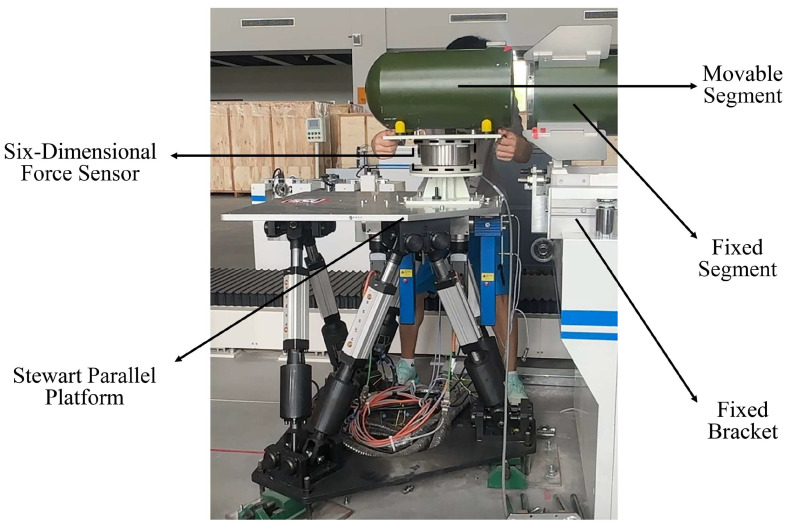
Stewart human-robot collaborative parallelism platform.

This paper proposes a framework wherein the Stewart parallel platform collaborates with the operator to accomplish the cabin segment docking task. Within this framework, the operator exerts his expertise to guide the robot’s actions by applying force. The setup, depicted in Figure 1, consists of a fixed cabin segment fastened on a bracket, a six-axis force sensor on the end-effector, and a movable cabin segment meant for docking positioned above. Under the operator’s guidance, the robot is tasked with moving the movable cabin segment for assembly between different working points. The assembly task can be divided into two subtasks. The first is the initial mobile phase, where the robot requires swift movement. When nearing the fixed cabin segment, the operator applies minimal force, expecting precise movements from the robot, and slight orientation adjustments are required to ensure assembly accuracy. During the entire docking process, if the operator tries to counteract additional movements caused by inertial forces by applying opposing force, the robot should promptly respond with the corresponding reverse motion.

**Figure 2:**
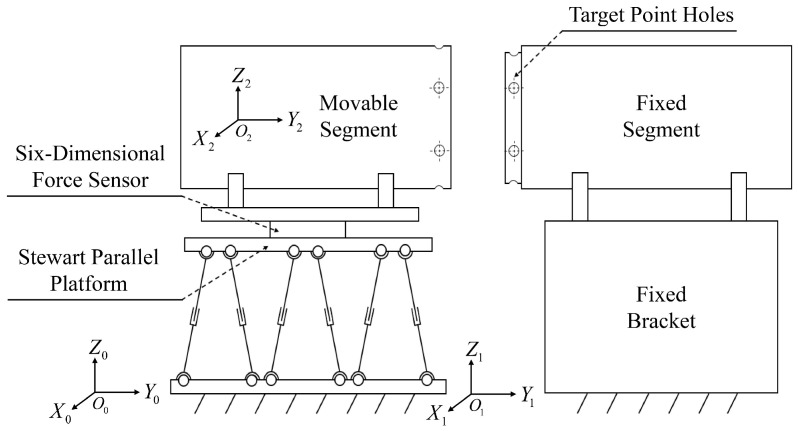
Schematic diagram of horizontal docking of compartments.

**Figure 3:**
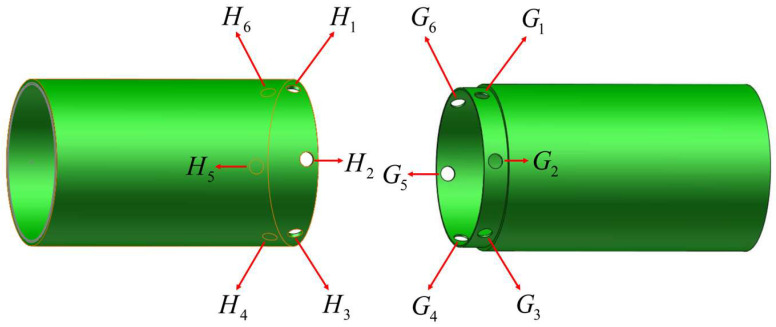
Schematic of the target point on the end face of the segment.

As illustrated in Figure 2, the cabin segment human-robot collaboration docking system encompasses the Stewart parallel platform, fixed bracket, movable cabin segment, and fixed cabin segment, among other components. The pivotal Stewart parallel platform serves as the mechanism to control and adjust the posture of the movable cabin segment. This comprises an active platform, a fixed platform, support chains, servo motors, drivers, force sensors, and industrial control computers. To aid the operator in accomplishing the docking task, target point positions and coordinate systems are defined across different components of the docking system, as depicted in Figure 2 and Figure 3. The definitions of coordinate systems and key points are outlined as follows:

(1) Geodetic coordinate system (base), denoted as $O_{0}-X_{0}Y_{0}Z_{0}$;
(2) The coordinate system firmly linked to the static platform of the parallel mechanism (hereafter called the static coordinate system), noted as $O_{1}-X_{1}Y_{1}Z_{1}$;
(3) The coordinate system firmly connected to the moving platform of the parallel mechanism (hereinafter termed the moving coordinate system), denoted as $O_{2}-X_{2}Y_{2}Z_{2}$;
(4) The docking points for the movable segments include the following: $H_{1}$, $H_{2}$, $H_{3}$, $H_{4}$, $H_{5}$, $H_{6}$;
(5) The docking points for the fixed compartment segments consist of the following: $G_{1}$, $G_{2}$, $G_{3}$, $G_{4}$, $G_{5}$, $G_{6}$.

## 3. Control Design

**Figure 4:**
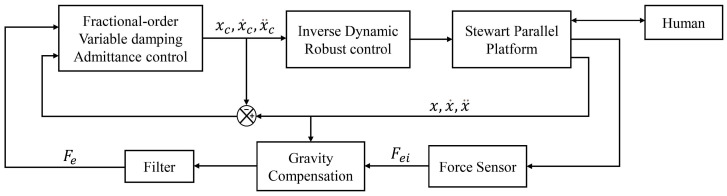
Block diagram of a guide frame-controlled robot. The control system consists of a variable damped admittance control generating a compliance trajectory and a robust motion control system.

This paper proposes a fractional-order variable damping admittance control (FVDAC) and an inverse dynamics robust control, facilitating highly compliant, real-time, and stable human-parallel robot collaboration specifically tailored to aerospace cabin segment docking. The proposed control schemes are illustrated in Figure 4.

FVDAC aims to dynamically adjust the robot’s movements in real-time during collaboration, thereby averting collisions between cabin segments. This approach ensures high compliance and real-time collaboration in human-parallel robot collaboration. Throughout the collaboration, this controller receives interaction forces and real-time poses from the force sensor and Stewart parallel platform, producing an ideal compliant movement plan $\left(x_{c},{\dot{x}}_{c},{\ddot{x}}_{c}\right)$ in Cartesian coordinates. It contains a variable damping matrix, adapting damping parameters in response to the operator-applied interaction forces. Additionally, fractional-order control is used to enhance the controller’s responsiveness.

To prevent the unknown interaction forces during collaboration from affecting the motion accuracy of the Stewart platform, and consequently the assembly accuracy, this paper proposes an inverse dynamics robust control. This method ensures highly stable human-parallel robot collaboration. This controller formulates a stable control law to align the robot’s end-effector motion state $\left(x,\dot{x},\ddot{x}\right)$ with the motion plan $\left(x_{c},{\dot{x}}_{c},{\ddot{x}}_{c}\right)$ devised by FVDAC. Furthermore, to account for unknown interaction forces affecting the end-effector and potential errors in the assembly scenario, it incorporates a nonlinear robust control.

### 3.1. Fractional-Order Variable Damping Admittance Control

To prevent collisions between cabin segments during docking, real-time adjustments to the robot’s motion are imperative. This requires the Stewart platform to exhibit high compliance and rapid responsiveness to interaction forces. While admittance control schemes are prevalent in most HRC studies, their conventional use primarily focuses on avoiding additional energy from affecting the system’s controllers. This limitation makes traditional admittance control unable to meet the real-time responsiveness and compliance demands of robots during collaboration [23]. Hence, improving traditional admittance control is necessary.

The goal of traditional admittance control is to make the robot behave like a specific mass-spring-damping system, enabling it to promptly react to the forces applied by the operator and devise a motion plan based on these external forces. The standard law for classical admittance control is given as follows:

(1) $$M_{D}(\ddot{x}-{\ddot{x}}_{c})+K_{D}(\dot{x}-{\dot{x}}_{c})+K_{P}(x-x_{c})=Q_{i}$$

where $\left(x,\dot{x},\ddot{x}\right)$ is the state of motion when no external force is applied, $M_{D}$, $K_{D}$, $K_{P}$ are the inertia, damping, and stiffness matrices in the Cartesian coordinate system, and $Q_{i}$ is interaction force during human-computer collaboration. The Cartesian end-position of the robot is denoted by the unit quaternion $q=\{q_{\eta },q_{\varepsilon }\}$, and $q_{\eta }$ is the scalar part of q and $q_{\varepsilon }$ is the vector part.

The interplay between admittance-controlled motion output and force input relies heavily on admittance parameters. Hence, selecting suitable admittance parameters is crucial in HRC. In many HRC scenarios, stiffness matrices and predefined motion plans are often excluded, offering full control of the robot’s actions to the operator. From the operator’s perspective, the velocity influenced by the damping value, inversely related to the controller’s planned velocity, significantly impacts the collaboration’s compliance. This paper proposes variable damping admittance control, which is designed to dynamically adjust adduction control parameters based on the external forces encountered during collaboration. To achieve this, a function governing damping is introduced, as follows:

(2) $$\alpha =tanh(Q_{i}^{T}Q_{i})$$

The function’s value is limited within the range [0, 1), where it equals 0 in the absence of force in any direction and incrementally approaches 1 with the gradual increase in interaction force.

In assembly collaborative tasks, an operator’s intention is commonly classified into swift movements and precise adjustments. The operator will exert a larger interaction force when he seeks a swift motion. The robot should reduce damping to augment motion speed, facilitating quicker task accomplishment. On the other hand, when the operator finetunes the position of the movable cabin segment, the robot should elevate damping to diminish motion speed, facilitating the finetuning operation being conducted by the operator. To accomplish this, a variable matrix is suggested as the damping matrix:

(3) $$K_{D}(\alpha )=(1-\alpha )K_{D_{c}}$$

where $K_{D_{c}}$ is the damping matrix with fixed values; $K_{D}(\alpha )$ is the damping matrix at the time of collaboration $K_{D_{c}}$ adjusted to the value of variant α.

Although the previously described variable damping parameter strategy aids the robot in achieving compliance during the collaboration process, the controller experiences transient overshoot and has a slow tracking response. The inherent memory characteristic of fractional-order derivatives can enhance the transient response of the control system, thereby enhancing the closed-loop system’s performance. Mathematically, various definitions of fractional-order derivatives exist [24]. This paper is mainly concerned with the solution of fractional-order differential equations using the Laplace transform, so the Caputo definition is used in this paper. The Caputo derivative of any order is computed using Formula (4):

(4) Dtp0Cf(t)=Δ1Γ(n−p)∫0t(t−τ)n−p−1f(n)(τ)dτ,(0≤n−1<p<n,n∈N)

The Laplace transform corresponding to the Caputo derivative of any order is represented by the following equation:

(5) $$\begin{array}{l} L{\{}_{0}^{C}D_{t}^{p}f(t);s\}=L{\{}_{0}^{RL}D_{t}^{p-n}f^{\left(n\right)}(t);s\}=s^{p-n}G(s)\\ =s^{p-n}(s^{n}Q(s)-\sum_{k=0}^{n-1}s^{n-k-1}x_{e}^{\left(k\right)}(0))\\ =s^{p}Q(s)-\sum_{k=0}^{n-1}s^{p-k-1}x_{e}^{\left(k\right)}(0),(n-1<p<n) \end{array}$$

Replacing the integer order in Equation (1) with a fractional order yields a fractional-order variable damped admittance control (FVDAC) on a single degree of freedom.

(6) MDDtβC(xe)+KD(α)DtγC(xe)=Qi,(1<β<2,0<γ<1)

The simultaneous Laplace transformation of both sides of Equation (6) yields the following:

(7) $$M_{D}s^{p}X_{e}(s)+K_{D}(\alpha )s^{q}X_{e}(s)=M_{D}s^{p-1}x_{e}(0)+M_{D}s^{p-2}{\dot{x}}_{e}(0)+K_{D}(\alpha )s^{q-1}x_{e}(0)+Q(s)$$

The collation leads to the following:

(8) $$\begin{array}{l} X_{e}(s)=\frac{M_{D}s^{p-1}x_{e}(0)+M_{D}s^{p-2}{\dot{x}}_{e}(0)+K_{D}(\alpha )s^{q-1}x_{e}(0)+Q(s)}{M_{D}s^{p}+K_{D}(\alpha )s^{q}}\\ =\frac{M_{D}s^{p-1}x_{e}(0)}{M_{D}s^{p}+K_{D}(\alpha )s^{q}}+\frac{M_{D}s^{p-2}{\dot{x}}_{e}(0)}{M_{D}s^{p}+K_{D}(\alpha )s^{q}}+\frac{K_{D}(\alpha )s^{q-1}x_{e}(0)}{M_{D}s^{p}+K_{D}(\alpha )s^{q}}+\frac{Q(s)}{M_{D}s^{p}+K_{D}(\alpha )s^{q}} \end{array}$$

The Mittag-Leffler function finds extensive use in fractional-order calculus and differential equations due to its analytic nature in the complex domain, allowing for infinite series expansions that aid in solving fractional-order differential equations. The definition of the multivariate Mittag-Leffler function (in the n-dimensional case) is as follows:

(9) $$E_{(a_{1},\cdots ,a_{n}),b}(z_{1},\cdots ,z_{n})=\sum_{k=0}^{\infty }\sum_{l_{1}+\cdots +l_{n}=k}\frac{k!}{l_{1}!\times \cdots \times l_{n}!}\frac{\prod_{i=1}^{n}z_{\;i_{i}}^{l}}{\Gamma (b+\sum_{i=1}^{n}a_{i}l_{i})}$$

where $b>0,a_{i}>0,\left|z_{i}\right|<\infty ,l_{i}>0,i=,\cdots ,n$.

Specifically, for n=1, the unitary Mittag-Leffler function is commonly utilized:

(10) $$E_{a_{1},b}(z_{1})=\sum_{k=0}^{\infty }\frac{z_{k}^{1}}{\Gamma (b+ka_{1})}\;a_{1},b>0,\left|z_{1}\right|<\infty$$

The Laplace transform of the Mittag-Leffler function is given by the following equation:

(11) $$L\{t^{\alpha k+\beta -1}E_{\alpha ,\beta }^{\left(k\right)}(\pm at^{\alpha });s\}=\int_{0}^{\infty }e^{-st}t^{\alpha k+\beta -1}E_{\alpha ,\beta }^{\left(k\right)}(\pm at^{\alpha })dt=\frac{k!s^{\alpha -\beta }}{{\left(s^{\alpha }\mp a\right)}^{k+1}},Re(s)>{\left|a\right|}^{\frac{1}{a}}$$

The Mittag-Leffler function can be used to describe the fractional-order differential equation presented above, and then, according to the Laplace transform of the Mittag-Leffler function, the following inverse transformation can be obtained:

(12) $$L^{-1}\{\frac{M_{D}s^{p-1}x_{e}(0)}{M_{D}s^{p}+K_{D}(\alpha )s^{q}};s\}=\frac{M_{D}}{K_{D}(\alpha )}x_{e}(0)t^{q-p}E_{q-p,q-p+1}(-\frac{M_{D}}{K_{D}(\alpha )}t^{q-p})$$

(13) $$L^{-1}\{\frac{M_{D}s^{p}{\dot{x}}_{e}(0)}{M_{D}s^{p}+K_{D}(\alpha )s^{q}};s\}=\frac{M_{D}}{K_{D}(\alpha )}{\dot{x}}_{e}(0)t^{q-p+1}E_{q-p,q-p+2}(-\frac{M_{D}}{K_{D}(\alpha )}t^{q-p})$$

(14) $$L^{-1}\{\frac{K_{D}(\alpha )s^{q-1}x_{e}(0)}{M_{D}s^{p}+K_{D}(\alpha )s^{q}};s\}=\frac{K_{D}(\alpha )}{M_{D}}x_{e}(0)t^{p-q}E_{p-q,p-q+1}(-\frac{K_{D}(\alpha )}{M_{D}}t^{p-q})$$

(15) $$L^{-1}\{\frac{Q(s)}{M_{D}s^{p}+K_{D}(\alpha )s^{q}};s\}=Q_{i}*(\frac{1}{K_{D}(\alpha )}t^{q-1}E_{q-p,q}(-\frac{M_{D}}{K_{D}(\alpha )}t^{q-p}))$$

Then, the analytical solution of the control system can be obtained using the above equations.

(16) $$\begin{array}{l} x_{e}=\frac{M_{D}}{K_{D}(\alpha )}x_{e}(0)t^{q-p}E_{q-p,q-p+1}(-\frac{M_{D}}{K_{D}(\alpha )}t^{q-p})+\frac{M_{D}}{K_{D}(\alpha )}{\dot{x}}_{e}(0)t^{q-p+1}E_{q-p,q-p+2}(-\frac{M_{D}}{K_{D}(\alpha )}t^{q-p})\\ +\frac{K_{D}(\alpha )}{M_{D}}x_{e}(0)t^{p-q}E_{p-q,p-q+1}(-\frac{K_{D}(\alpha )}{M_{D}}t^{p-q})+Q_{i}*(\frac{1}{K_{D}(\alpha )}t^{q-1}E_{q-p,q}(-\frac{M_{D}}{K_{D}(\alpha )}t^{q-p})) \end{array}$$

Fractional-order variable damping admittance control introduces parameters β and γ compared to integer-order variable damping admittance control, allowing for better adjustment of the dynamic interactive behavior between the robot and interaction forces, and thus making the control strategy design more flexible. When the orders β and γ both take their minimum values, this becomes first-order damping control, and when the order β and γ both take their maximum values, this becomes second-order integer admittance. Thus, it is understood that, with decreasing orders, the fractional-order characteristics gradually diminish the inertial storage energy, while the energy-dissipation characteristics will take precedence. The specific orders of the controller can be determined based on this characteristic.

### 3.2. Robust Motion Control

In admittance control, velocity and acceleration in motion planning are typically time-varying functions. To achieve motion planning generated by fractional-order variable damping admittance control on the Stewart platform, inverse dynamic control must be utilized. The inertia and Coriolis terms in the dynamic model of the Stewart platform remain uncertain due to the presence of unknown interaction forces. Therefore, the robustness of the inverse dynamics control is necessary to ensure that the Stewart platform maintains an accurate tracking performance for the supple trajectories generated by the fractional-order conductor control, even in the presence of unknown interaction forces, and to satisfy the stringent requirements for segmental docking accuracy. The expression of Lagrangian dynamics equations emerges when an external force, $h_{e}$, acts upon the end of the Stewart parallel platform:

(17) $$M(l)\ddot{l}+C(l,\dot{l})\dot{l}+n(l,\dot{l})=u_{c}-J^{T}(l)h_{e}$$

The length l of each branch motorized cylinder is chosen as a generalized coordinate, M(l) is the inertia matrix of the Stewart parallel robot, $C(l,\dot{l})$ is the Coriolis force term, and $n(l,\dot{l})$ is an n-dimensional vector dependent on the state of the system (including nonlinear coupling terms such as the centripetal force, friction, and gravity), while $u_{c}$ is a control input term, which is modeled as a nonlinear multivariate control system. The following control law can be obtained after compensating for the nonlinearity:

(18) $$u_{c}=\hat{M}(l)y+\hat{C}(l,\dot{l})\dot{l}+\hat{n}(l,\dot{l})+J^{T}(l)h_{e}$$

where $\hat{M}(l)$, $\hat{C}(l,\dot{l})$ and $\hat{n}(l,\dot{l})$ are the estimated values of M(l), $C(l,\dot{l})$ and $n(l,\dot{l})$, respectively, and y is the control input to be designed for the desired motion planning. A common approach is to choose the following input, as described in [25]:

(19) $$y=\bar{J}(l)(\ddot{\tilde{x}}+K_{D_{t}}\dot{\tilde{x}}+K_{P_{t}}\tilde{x}-\dot{J}(l,\dot{l})\dot{l})$$

where $K_{D_{t}}$ and $K_{P_{t}}$ are both positive definite diagonal matrices, $\bar{J}(l)$ is the generalized inverse matrix of the Jacobi matrix, and $\tilde{x}=x_{c}-x$ denotes the position error. Substituting (19) into (18), and then substituting (18) into (17), the closed-loop dynamics model is obtained as follows:

(20) $$\ddot{l}=y-[I-M^{-1}(l)\hat{M}(l)]y+M^{-1}(l)[\tilde{C}(l,\dot{l})\dot{l}+\tilde{n}(l,\dot{l})]$$

where $\tilde{C}(l,\dot{l})=\hat{C}(l,\dot{l})-C(l,\dot{l})$, $\tilde{n}(l,\dot{l})=\hat{n}(l,\dot{l})-n(l,\dot{l})$.

Take y in Equation (19) as the control input and set the following:

(21) $$\eta^{*}=J[I-M^{-1}\hat{M}][y+{\hat{M}}^{-1}(\tilde{C}\dot{l}+\tilde{n})]-J{\hat{M}}^{-1}(\tilde{C}\dot{l}+\tilde{n})$$

Then

(22) $$\begin{array}{l} \eta^{*}=[\ddot{\tilde{x}}+K_{D_{t}}\dot{\tilde{x}}+K_{P_{t}}\tilde{x}-\dot{J}\dot{l}]+J[I-M^{-1}\hat{M}]\hat{M}+(\tilde{C}\dot{l}+\tilde{n})+J\ddot{l}\\ =\ddot{\tilde{x}}+K_{D_{t}}\dot{\tilde{x}}+K_{P_{t}}\tilde{x}-\dot{J}\dot{l}-J\ddot{l} \end{array}$$

It is clear that $\tilde{x}$ is no longer guaranteed to converge to zero due to the presence of $\eta^{*}$. Therefore, it is necessary to study the stability of the controller under unknown external forces in depth. To ensure the robustness of the control law, it is now considered that a control term ω is added to expression (19), and (19) becomes the following:

(23) $$y=\bar{J}(l)(\ddot{\tilde{x}}+K_{D_{t}}\dot{\tilde{x}}+K_{P_{t}}\tilde{x}-\dot{J}(l,\dot{l})\dot{l}+\omega )$$

To determine the exact value of ω, Lyapunov’s second method was used to determine the stability of the system, the following positive definite quadratic form is selected as the Liapunov function:

(24) $$V(\xi )=\xi^{T}A\xi >0$$

where $\xi ={\left[\tilde{x}\dot{\tilde{x}}\right]}^{T}$ is the state variable of the system, A is a positive definite symmetric matrix, while the derivative of the function V concerning time is as follows:

(25) $$\begin{array}{l} \dot{V}={\dot{\xi }}^{T}A\xi +\xi^{T}A\dot{\xi }\\ =\xi^{T}(H^{T}A+AH)\xi +2\xi^{T}AD(\eta^{*}+\dot{J}\dot{l}+J\ddot{l}-\omega )\\ =-\xi^{T}P\xi +2\xi^{T}AD(\eta^{*}+\dot{J}\dot{l}+J\ddot{l}-\omega ) \end{array}$$

where $P=-(H^{T}Q+QH)$ is a symmetric matrix, $H=\left[\begin{array}{cc} 0 & I\\ -K_{P_{t}} & -K_{P_{t}} \end{array}\right]$, $D=\left[\begin{array}{l} 0\\ I \end{array}\right]$. 

To ensure asymptotic stability, a must satisfy $\dot{V}<0$. The first term on the right-hand side of the equal sign in (25) is negatively definite, so ω is taken to ensure only that the second term on the right-hand side of the equal sign is less than or equal to zero. Set $z=2D^{T}A\xi$ and take ω to be the following value:

(26) $$\omega =\frac{\rho }{\left\|z\right\|}Z$$

Due to the kinematic constraints of a robot, the following reasonable assumptions can be made:

(27) $$\left\|\dot{J}\dot{l}\right\|\leq \beta <\infty ,\forall l,\dot{l}$$

(28) $$\left\|J\ddot{l}\right\|\leq f<\infty ,\forall l,\dot{l}$$

where ρ is a positive scalar; then, the second term on the right-hand side of the equality sign of (25) becomes the following:

(29) $$\begin{array}{l} z^{T}(\eta^{*}+\dot{J}\dot{l}+J\ddot{l}-\frac{\rho }{\left\|\left.z\right\|\right.}z)=z^{T}(\eta^{*}+\dot{J}\dot{l}+J\ddot{l})-\rho \left\|\left.z\right\|\right.\\ \leq \left\|\left.z\right\|\right.(\left\|\left.\eta^{*}\right\|\right.+\beta +f)-\rho \left\|\left.z\right\|\right.=\left\|\left.z\right\|\right.(\left\|\left.\eta^{*}\right\|\right.+\beta +f-\rho ) \end{array}$$

The value of ρ must be satisfied:

(30) $$\rho >\left\|\eta^{*}\right\|+\beta +f$$

While the uncertainty term $\eta^{*}$ remains unknown, the introduction of the control term ω ensures that $\dot{V}$ is a negative definite. 

Finally, within the sliding subspace of z=0(A=0), the ideal theoretical scenario is for the control signal ω to operate at infinite frequency [26]. Nevertheless, in practice, this signal experiences high-frequency alternations, resulting in torque oscillations. To mitigate this high-frequency exchange (jitter), a specific range ε, adjacent to the sliding subspace, is defined. Subsequently, the control law that was proposed earlier is modified based on this range as follows:

(31) $$\omega =\left\{\begin{array}{lll} \frac{\rho }{\left\|z\right\|}Z & \mathrm{for} & \left\|z\right\|\geq \varepsilon \\ \frac{\rho }{\varepsilon }Z & \mathrm{for} & \left\|z\right\|<\varepsilon \end{array}\right.$$

The fractional-order variable damping admittance control (FVDAC) and the inverse dynamics robust control presented in this section form a tandem relationship in the control system. The FVDAC acts as an upper-level decision algorithm designed to generate a compliant motion plan based on the interaction forces in human-robot collaboration. Then, the inverse dynamics robust control receives this motion plan and controls the parallel robot to execute this compliant motion plan with stability and precision.

## 4. Experimental Verification

### 4.1. Experimental Settings

Fractional-order control and robust motion control experiments were performed on the most heavily loaded degree of freedom translating along the *Z*-axis. Following this, an assembly scenario, exemplified by a docking experiment, was used to illustrate the impact of the variable damping strategy. A LabView program communicated with the robot via UDP protocol, operated with a 5 ms sampling period. An external computer monitored the robot’s status, facilitating gravity compensation for the six-axis force sensor and execution of the control laws outlined in Section 2 to generate a compliant motion plan. Finally, utilizing the motion control programs built on the dynamics of the Stewart robot, the necessary driving forces for the six-branch electric cylinders were regulated. The parameters for admittance control law (1) are as follows:

$$M_{D}=diag_{6}\{2,2,2,0.3,0.3,0.3\},\;K_{D_{c}}=diag_{6}\{5,5,5,10,10,10\},\;K_{P_{c}}=diag_{6}\{0\}.$$

The following parameters were determined through repeated debugging to achieve a fast and stable response. The value of the order in Equation (5) was taken as: β=1.2, γ=0.9. For robust motion controllers, the matrix was selected as: $K_{D_{t}}=diag_{6}\{10\}$, $K_{P_{t}}=diag_{6}\{0\}$, with the selection of parameters corresponding to nonlinear robust motion control ρ=50

$$A=\left[\begin{array}{l} A_{11}A_{12}\\ A_{21}A_{22} \end{array}\right]$$

where $A_{11}=diag_{6}\{6.334\}$, $A_{12}=diag_{6}\{3.666\}$, $A_{21}=A_{12}$, $A_{22}=diag_{6}\{0.014\}$.

The props and simulated loads collectively form a unified entity, which can be regarded as a cuboidal mass, with mass $m_{B}$ = 52.68 kg and dimensions $lm=(l_{B_{x}},l_{B_{y}},l_{B_{z}})$, and inertia $I_{B}=(I_{B_{xx}},I_{B_{yy}},I_{B_{zz}})$, as outlined in Table 1. The initial sensor signals were subjected to zero-point zero-drift processing and real-time gravity compensation, as in the literature [27], and then the noisy signals were filtered with a low-pass Butterworth filter.

### 4.2. Single-Degree-of-Freedom Experiment

**Figure 5:**
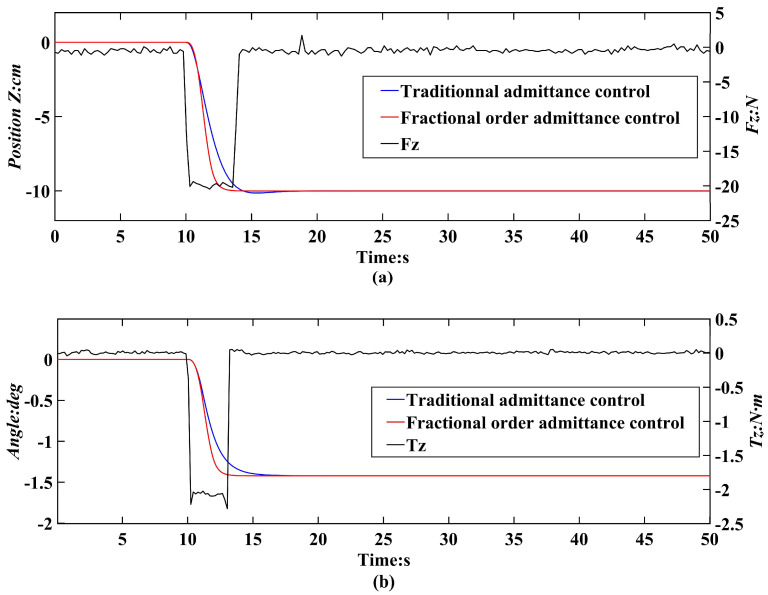
Tracking curves for dynamics control and dynamics robust control. (**a**) Comparison of response performance in three translational degrees of freedom along the *Z*-axis as an example of verification. (**b**) Comparison of the response performance in the three rotational degrees of freedom, verified using the rotational degree of freedom around the *Z*-axis as an example.

The effectiveness of the fractional-order admittance control was evaluated on individual degrees of freedom. Due to the different parameter settings for translational and rotational degrees of freedom, experiments were conducted on both Cartesian translational and rotational degrees of freedom. Figure 5 shows a response performance comparison between the conventional admittance control and the fractional-order admittance control. Figure 5a,b show the position/attitude change curves of the Stewart parallel platform under the control of the two controllers after applying a force of Fz = 20 N and a moment of Tz = 2 Nm to the translational and rotational degrees of freedom of the *Z*-axis, respectively.

By comparing the curves, it was observed that, in the translational degree of freedom, applying a force of 20 N for 3 s led to a 52.05% increase in response speed and a slight reduction in overshoot when employing fractional-order admittance control. Similarly, during the rotational degree of freedom, applying a torque of 20 Nm for 3 s showcased a 41.28% higher response speed in fractional-order admittance control in contrast to traditional admittance control. These comparative outcomes distinctly highlight the substantial enhancement in response speed achieved through fractional-order admittance control as opposed to traditional methods. Subsequent assembly experiments have shown that this improvement adequately meets the requirement for a fast robot response during cabin segment docking.

**Figure 6:**
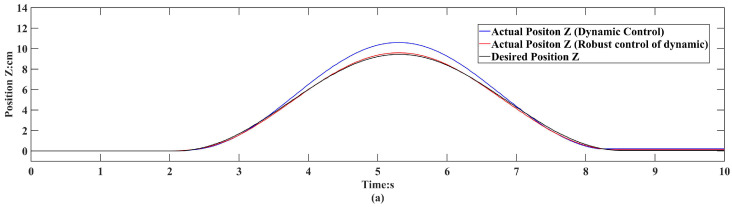

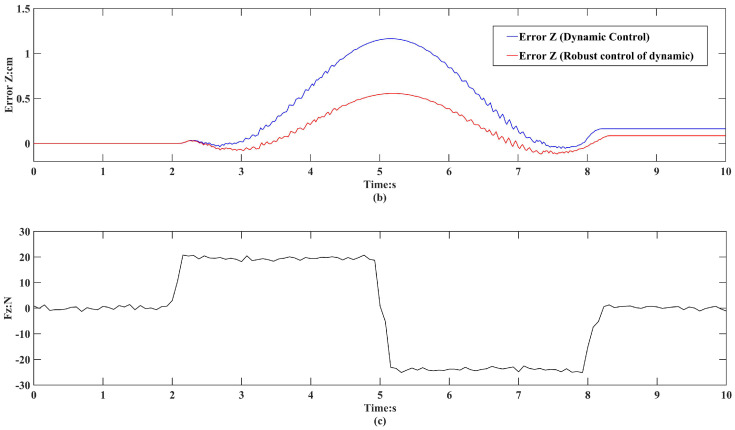
Tracking error for dynamics control and dynamics robust control. (**a**) Comparison of the tracking of the desired position profile of a Stewart parallel platform in the *Z*-axis translational degree of freedom under dynamics control versus dynamics robust control. (**b**) Comparison of tracking error of desired position profile by Stewart parallel platform in *Z*-axis translational degree of freedom under dynamics control vs. dynamics robust control. (**c**) The force applied to the degrees of freedom of the *Z*-axis in the experiment.

The robustness of the inverse dynamic robust controller against unknown external forces underwent validation via experiments. The experimental results are shown in Figure 6. Tests encompassed both simple dynamic control (Equation (14)) and the resultant inverse dynamics robust control. These experiments entailed applying a 20 N upward force along the *Z*-axis for 3 s, followed by a downward force of 20 N on the end-effector of the parallel robot.

Upon the generation of interaction forces, it becomes apparent that the tracking error of the controllers gradually increases. Around 5.3 s, both the inverse dynamics controller and the inverse dynamics robust controller contained peak tracking errors. Specifically, the peak tracking error for the inverse dynamic controller was 1.18 cm, whereas for the inverse dynamic robust controller, this remained at only 0.57 cm. In comparison to the simple dynamic controller, the dynamic robust controller consistently produces smaller, bounded errors. Conversely, the simple dynamic controller yields larger errors, potentially causing unexpected robot motion during interaction. Through this experiment, the necessity of introducing robust control in dynamic controllers for achieving high-precision segment docking and the robustness of the proposed controller against unknown external forces are demonstrated.

### 4.3. Assembly Experiment

**Figure 7:**
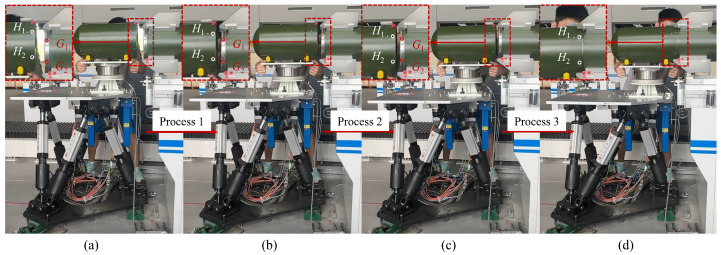
Schematic illustration of the operational phases of human-robot collaboration for segment docking: (**a**) The robot is in the starting position, and human-robot collaboration begins. (**b**) The operator guides the robot to move the movable segment near the fixed segment. (**c**) The operator compares the target points’ relative positions and adjusts the active segment’s attitude. (**d**) Segment docking mission completed.

The proposed method underwent evaluation in a practical scenario involving missile cabin assembly production. The experimental setup included placing a warhead model on the Stewart platform’s end-effector. The assembly process necessitated collaborative movement by both the operator and the robot: initially shifting the warhead model from its initial position towards the fixed part, then precisely adjusting its position and orientation using pre-marked target points before completing the docking. The robot’s movement comprised three stages: primarily movement in the first stage, precise fine-tuning of position and attitude in the second, and subsequent movement in the third stage. Figure 7 illustrates the distinct states of the robot throughout this collaborative task, comprising the starting, movement, adjustment, and ending states. This experiment further validates the advantages and practical applicability of the proposed fractional variable damping admittance controller and inverse dynamical robust controller in actual cabin docking scenarios. The various states of the robot in the collaborative task are presented in the following.

**Figure 8:**
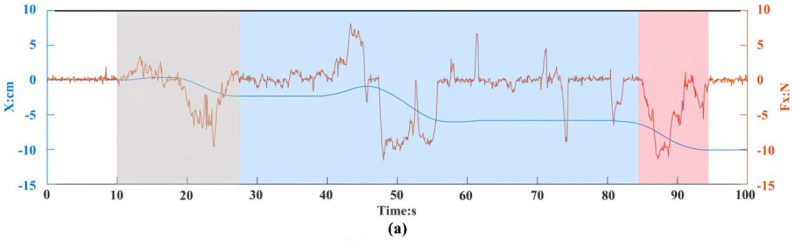

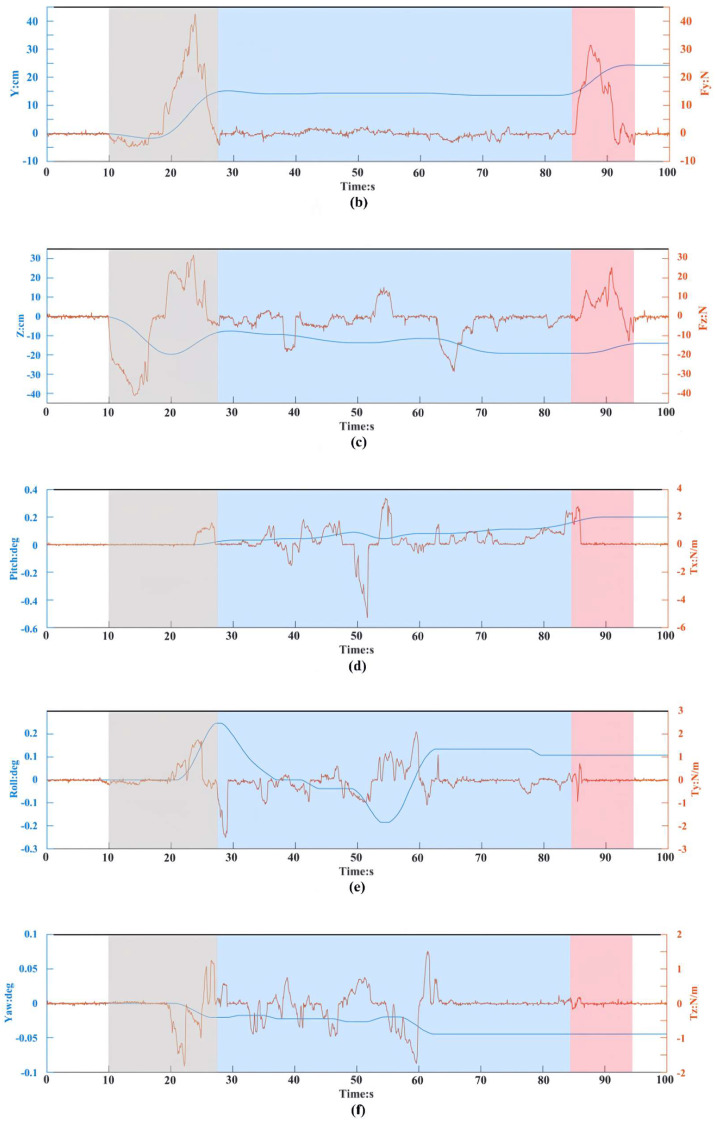
Robot position change and interaction force in collaborative experiments. (**a**–**c**) show the interactive force along the X, Y, and Z axes of translational degrees of freedom and the position curve of the robot in the assembly process respectively. (**d**–**f**) show the interaction torque and the position curve of the robot on the degrees of freedom of rotation around the X, Y and Z axes in the assembly process, respectively. The red curve is the interaction force, and the blue curve is the position curve of the robot.

Figure 8 illustrates the comprehensive record of the robot’s pose and interaction forces throughout the entire experiment. The interaction between the operator and the robot unfolds in three distinct processes.

Process 1: Characterized by the manual guidance of the robot towards the vicinity of the fixed cabin segment, denoted by a gray background.

Process 2: involving fine manual adjustments of the robot, highlighted in light blue.

Process 3: encompassing the manual guidance for docking, represented by a faint red color.

In process 1 (from 10 s to 27 s), the operator primarily guided the robot in the three translational degrees of freedom. Let us focus on Figure 8c, from 10 s to 17 s, where the operator applied a peak negative interaction force of 40 N, resulting in the robot moving at a velocity of 1.83 cm/s; subsequently, from 17 s to 27 s, the operator applied a peak positive interaction force of 30 N, causing the robot to move at a velocity of 1.67 cm/s. This behavior mirrors real-world scenarios where the operator counteracts inertial forces, prompting an immediate reverse movement of the robot.

In process 2 (from 27 s to 84 s), also focus on Figure 8c, where from 27 to 38 s, the operator applies several negative interaction forces, peaking around 5 N, resulting in velocities of approximately 0.089 cm/s. A comparison between the robot’s behavior in the *Z*-axis from 10 s to 17 s reveals the impact of introducing variable damping coefficients: the robot promptly adjusts its motion speed in response to the interaction force, aligning with expectations thanks to variable damping coefficients that offer advantages during this stage. When the operator applies a smaller interaction force, the admittance control’s damping coefficient increases, slowing down the robot’s movement. This allows the operator to feel the robot become firmly attached to their hand, enabling the operator to make adjustments promptly based on the situation. The performance of the robot in the translational degree of freedom in the *Z*-axis is illustrated above, because this direction carries the heaviest loads. It can be seen that FVDAC has the same effect on the other degrees of freedom by analyzing other curves.

In Process 3 (from 84 s to 94 s), the motion is mainly along the three translational degrees of freedom. It can be seen that the movement profile shows slight fluctuations in the other degrees of freedom (e.g., from 84 s to 86 s, the robot undergoes a small amount of rotational motion along with translational motion), which is due to the operator applying unregulated external forces throughout the process. No special attention is needed, however, as these fluctuations represent the operator’s intent.

Experiments confirm that the proposed variable damping matrix, which allows for the robot to regulate its motion speed according to the interaction forces, can be used in practical applications to realize fast motion, precise motion, or reverse motion during the assembly process, thus preventing collisions caused by inertial forces while accomplishing the segment docking task. Moreover, this approach is also potentially applicable in other similar scenarios involving robots and humans collaborating in assembly lines.

## 5. Conclusions

This study introduces a novel approach for human-robot collaboration to ensure the collision-free docking of compartment segments using parallel robots. The proposed method achieves highly compliant, real-time and stable human-robot collaboration on a Stewart parallel platform. The method innovatively incorporates a variable damping matrix and fractional-order control into the traditional admittance control to realize dynamic motion velocity adjustment based on interaction forces. In addition, an inverse dynamics robust controller for the Stewart parallel platform human-robot collaboration is developed to ensure stable motion control even in the presence of unknown interaction forces for precise aerospace segment docking requirements. The proposed fractional-order variable damping admittance control, as well as the inverse dynamics robust control, are the focuses of experiments on the axial degree of freedom along the *Z*-axis, which is the heaviest loading task, and the experimental results show that the response speed of the admittance control algorithm is significantly improved after the introduction of the fractional-order derivative with the same interacting force, and the tracking errors of the motion controller is significantly reduced after the introduction of the proposed robust control constituent. Finally, the effect of the proposed fractional-order variable damping admittance control in practical applications is verified by completing the human-robot collaboration experiment focusing on the docking task. The experiment completed the segment docking task through three processes: position adjustment, attitude finetuning and guided docking. The high-speed motion for position adjustment, the low-speed motion for attitude finetuning, and the reverse motion for avoiding segment collision in real segment docking are well simulated. The results show that the proposed method enables the robot to have a high speed when subjected to large interaction forces, a low speed when subjected to small interaction forces, and to counteract inertial forces to immediately perform a reverse motion to avoid segment collisions when subjected to instantaneous and sudden changes in reverse forces. Future research will focus on optimizing the order selection in fractional-order control and inertia matrix tuning strategies, and further consider ergonomics-related factors such as operator fatigue and learning curves for more accurate heavy assembly tasks.

## Figures and Tables

**Table 1:** Mass and geometric parameters of the load-bearing props.

	$l_{B_{x}}/mm$	$l_{B_{y}}/mm$	$l_{B_{z}}/mm$	$I_{B_{xx}}/kg\cdot mm$	$I_{B_{yy}}/kg\cdot mm$	$I_{B_{zz}}/kg\cdot mm$
Value	50	100	10	0.238	0.167	0.102

## Data Availability

Data are contained within the article.
